# Understanding does not depend on (causal) explanation

**DOI:** 10.1007/s13194-018-0240-6

**Published:** 2019-01-04

**Authors:** Philippe Verreault-Julien

**Affiliations:** Erasmus School of Philosophy, Erasmus University Rotterdam, P.O. Box 1738, 3000 DR Rotterdam, The Netherlands

**Keywords:** Understanding, Explanation, Models, Non-causal, How-possibly explanations

## Abstract

One can find in the literature two sets of views concerning the relationship between understanding and explanation: that one understands only if 1) one has knowledge of causes and 2) that knowledge is provided by an explanation. Taken together, these tenets characterize what I call the narrow knowledge account of understanding (narrow KAU). While the first tenet has recently come under severe attack, the second has been more resistant to change. I argue that we have good reasons to reject it on the basis of theoretical models that provide how-possibly explanations. These models, while they do not explain in the strict (narrow KAU) sense, afford understanding. In response, I propose an alternative epistemology of understanding, broad KAU, that takes cases of theoretical modelling into account.

## Introduction

The epistemology of understanding has traditionally been related, if not reduced, to the epistemology of causal explanation. A prominent view about scientific understanding (understanding hereafter) is that one understands a phenomenon if and only if one has knowledge of its cause(s). Pritchard (2014), from whom I borrow the terminology, calls this underlying epistemology of understanding the ‘knowledge account of understanding’. Jointly with the traditional requirement that only causal explanations can supply this knowledge, I characterize the combination of these two views as the *narrow* knowledge account of understanding (narrow KAU hereafter). Narrow KAU has two tenets: 1) causal knowledge is necessary for understanding and 2) only explanations can provide that knowledge.^1^ The qualifier ‘narrow’ emphasizes that within narrow KAU, only knowledge of causes is a source of understanding and that only explanations can provide that knowledge. In other words, it is narrow because it states that knowing a *causal explanation* is a necessary condition for understanding.

One important desideratum of an account of understanding is that it provides evaluative criteria to demarcate cases of genuine from illusory understanding. The sense of understanding (see Trout 2002; Ylikoski 2009) can be a misleading cue for genuine understanding, the state of actually understanding. As such, it must be recognized that *mis* understanding is a live possibility and that in consequence we must have criteria that are stringent enough.

However, a second important desideratum is that the account does not rule out large areas of science as not conducive to understanding. A naturalistic outlook on science should compel philosophers to not attribute systematic and persistent error across different fields of science (e.g. Hausman 2009, 40). While narrow KAU scores very well on the first desideratum, it does poorly on the second. It provides clear and explicit criteria for judging whether understanding is genuine, but at the cost of making illusory understanding dubiously prevalent. Indeed, as I shall argue, one implication of narrow KAU is that theoretical modelling often does not afford understanding because it does not provide explanations. An account of understanding with such implication is suspect.

As a matter of fact, an important motivation behind the burgeoning literature on non-causal explanation (e.g. Baker 2012; Baron et al. 2017; Batterman 2002; Batterman and Rice 2014; Lange 2013, 2017; Pincock 2015; Reutlinger 2014; Reutlinger and Saatsi 2018; Saatsi 2018) is to account for explanatory practices that are inconsistent with narrow KAU’s insistence on causal knowledge. According to this literature, we can gain understanding of empirical phenomena via explanations that do not rely on causal facts, therefore challenging narrow KAU.^2^ For instance, Pincock argues for a case of ‘abstract explanation’ on a basis that concurs with the second desideratum, namely that “[i]t is expert practitioners who should guide our judgements on cases and influence our philosophical theory of explanation” (2015, 870). According to him, abstract explanations work by classifying systems between which there are abstract dependence relations. Crucially, abstract explanations do not explain phenomena by virtue of identifying causal facts. For Pincock, causal and abstract explanations are hence of a different kind.

Since it is uncontroversial in the explanation and understanding literature that explanations afford understanding (e.g. de Regt 2009a; Grimm 2010; Khalifa 2017), cases of non-causal explanations provide persuasive counterexamples to narrow KAU’s first tenet. Even recent prominent accounts of causal explanation are now more liberal. For example, despite the fact that their main focus is on causal explanation, Woodward (2003, 220-221) and Strevens (2008, sec. 5.7) briefly indicate that the criteria they propose for explanatory relevance could also be applied to non-causal explanations.^3^ There is now a strong case for what Reutlinger (2016) calls the “liberal consensus”.^4^

The second tenet of narrow KAU has been more resistant to change. However, I believe it ought, like the first tenet, to be rejected because it does not satisfy the second desideratum. In the remainder of this paper, I first spell out in more details what the second tenet of narrow KAU amounts to. Then, I argue that both practitioners’ judgment as well as current philosophical accounts of so-called how-possibly explanations provide good reasons to abandon it. How-possibly explanations present a challenge to narrow KAU because they do not explain according to it, and yet appear to afford understanding. I will then propose what I call the *broad* knowledge account of understanding (broad KAU hereafter) that builds on a framework developed by Reutlinger (2016). Broad KAU, I hold, satisfies the two desiderata stated earlier and hence provides a normatively and descriptively compelling account of understanding.

## Narrow KAU and the necessity of explanation

If the first tenet of narrow KAU is more controversial, if not debunked, the second is more widely held. Proponents of this view consider that explanations are the only legitimate source of understanding, for instance:

The resulting objectivist, ontic, account, in generic form, states that scientific understanding is the state produced, and only produced, by grasping a true explanation (Trout 2007, 584-585).

[U]nderstanding amounts to (*a*) knowing that the explanans is true, (*b*) knowing that the explanandum is true, and (*c*) for some *l*, knowing that *l* is the correct explanatory link between the explanans and the explanandum (Khalifa 2012, 26).

An individual has scientific understanding of a phenomenon just in case they grasp a correct scientific explanation of that phenomenon (Strevens 2013, 510).^5^

In a nutshell, what these accounts say is that one can’t understand without an explanation. According to Trout, it is important to separate the sense of understanding, which can be a misleading phenomenology, from the genuine understanding one can only obtain when being in possession of a true explanation. Khalifa maintains that a distinct concept of understanding adds nothing to what he calls the “Explanatory Model of Understanding” (EMU). For Khalifa, having scientific understanding is a matter of having explanatory knowledge, i.e., highly virtuous beliefs about an explanation. Similarly, Strevens argues that what he designates as the “simple view” adequately depicts the connection between explanation and understanding. For him, the epistemology of explanation precedes and guides the epistemology of understanding. One understands *why* something is the case, according to Strevens, when one not only grasps a state of affairs, but also its correct explanation.

It is important to bear in mind that Strevens and Trout, in particular, do not claim that knowing an explanation is *sufficient* for understanding.^6^ Indeed, the ‘grasping’ condition may require a state or ability on top of knowledge. This is why Strevens (2013, 510) does not reduce understanding to explanation. However, grasping can be related to knowing. Indeed, as Strevens (2013, 513, fn. 6) indicates, his account is compatible with the view that knowledge is necessary for grasping. Trout is not as explicit as Strevens, but nevertheless suggests that one might “treat grasping as a kind of knowing” (2007, 585). In short, perhaps knowing an explanation is not sufficient—one may need to grasp it—, but to grasp a true explanation one may need to know it.^7^ That said, the important point is that one needs to stand in the appropriate epistemic relation—e.g., knowing, grasping, believing, etc.—with an explanation to understand. Explanations are the bearer of the information that affords understanding and without an explanation there is no understanding.

One consequence of these views is to downplay the import of understanding as a separate and useful notion. For instance, Khalifa (2012) argues that de Regt and Dieks’s (2005), de Regt’s (2009a, 2009b), and Grimm’s (2010) accounts of understanding do not add anything to our understanding of understanding on top of what EMU already says. If this is correct, then it indeed makes little sense to spell out the epistemic contribution of theoretical models in terms of understanding since explanation is in fact the key concept. However, Khalifa notes that one way Grimm could demonstrate the distinctiveness of understanding is “to argue that there are cases of understanding without explanation” (2012, 23). For if there can be understanding without explanation, then it means understanding can’t be fully reduced to explanation. It also means the epistemology of explanation may not provide all the normative and descriptive criteria we need when analysing science. Finding such cases thus holds the promise of rebutting the second tenet of narrow KAU’s and, more generally, to contribute to our understanding of the relationship between explanation and understanding.

## Is explanation necessary?

An explanation is essentially just a set of propositions that connects an explanans to an explanandum in the right way (Strevens 2013). It is uncontroversial that explanations provide the right kind of propositions and structure. Having said that, do we have good reasons to believe that knowing ‘non-explanatory’ propositions can’t afford understanding? Put differently, are there sets of propositions that convey information conducive to understanding, but that do not satisfy the conditions for explanation?

The strategy I follow here is similar to Lipton’s (2009).^8^ His strategy is to point out ways we can obtain the same cognitive benefits (e.g., knowledge of causes or of possibility) that explanations typically provide, but *without* having an explanation. According to him, understanding should therefore be identified with the benefits explanations provide rather than with the explanations themselves.

Similarly, I would like to show that one important source of knowledge conducive to understanding phenomena is studied in the philosophy of science literature under the label of ‘how-possibly explanations’ (Bokulich 2014; Craver 2006; Forber 2010; Grüne-Yanoff 2013a, 2013b; Rohwer and Rice 2013; Verreault-Julien 2017, 2018; Ylikoski and Aydinonat 2014). How-possibly explanations (HPEs hereafter) are to be contrasted with how-actually explanations (HAEs hereafter), or explanations simpliciter.

A terminological disclaimer is now essential. As their names suggest, it may seem that HPEs and HAEs are simply different species of the same genus ‘explanation’. Were it the case, then arguing as I shall do that having an explanation is not necessary because HPEs may afford understanding would be beside the point. For if having an HPE would amount to having an explanation, then it would say nothing about the necessity of explanation for understanding. So we first need to disentangle in what sense HPEs relate to the second tenet of narrow KAU.

Contemporary characterizations of HPEs are very similar to what Hempel called potential explanations (Bokulich 2014).^9^ According to him, a potential explanation is “any argument that has the character of a [deductive-nomological] explanation except that the sentences constituting its explanans need not be true” (Hempel 1965, 338). In HPEs, what constitutes the explanans is not true or not known to be true. Indeed, we may simply lack the appropriate justification for believing that a given HPE is, in fact, a HAE. HAEs, on the other hand, give a correct explanatory account of the explanandum. Another way of putting it is to say that HPEs satisfy internal conditions of adequacy—the explanation’s logical structure—whereas HAEs satisfy both internal and external—empirical correspondence to the world—ones (Strevens 2013).^10^ For instance, I could, using phlogiston theory, provide an internally correct explanation of the phenomenon of combustion. However, the theory does not actually explain combustion because there is no such entity as phlogiston. The explanation is false and does not meet the external conditions of adequacy.^11^ To give another example, Ptolemaic astronomy provided an internally correct explanation of the motion of the planets using a geocentric cosmology and epicycles. But since the theory is false because it depicts, among other things, the earth at the centre of the solar system and planets as moving along epicycles, it is not externally correct and thus not a HAE.

In practice, the difference between HPEs and HAEs is more like a spectrum.^12^ Sometimes we have some evidence or degree of confirmation that a HPE may be a HAE. HAEs may also be approximately true. Or HAEs can also vary in their explanatory power, scope, or breadth (see, e.g., Schupbach and Sprenger 2011; Woodward 2003; Ylikoski and Kuorikoski 2010). Strevens (2013, 513-514), in his defence of the necessity of explanation, also acknowledges that explanatory correctness comes in degree. But other times, we decidedly know that we only have a HPE, not a HAE. These are the cases that are of special interest for our consideration of the necessity of explanation.

For the purpose of my argument, what is important to bear in mind is that what narrow KAU requires for understanding is to have a HAE, not merely a HPE. The textual evidence of the previous section makes this plain. Strevens explicitly denies that potential explanations—or HPEs—afford understanding of phenomena. He indicates that grasping a correct explanation “requires grasping that the propositions expressing a relevant model’s explanatory content are true, or in other words, understanding that the states of affairs represented by those propositions obtain” (2013, 512). This requirement is clearly not satisfied by most cases of HPEs discussed in the literature since they depict either false—or not known to be true—explanantia or explananda. Their explanatory content is false and therefore one can’t, according to narrow KAU, reap understanding from them. The issue therefore does not hinge on whether HPEs are a species of explanation. The issue is rather whether a HAE is necessary for understanding. According to proponents of the second tenet of narrow KAU, it is.

## HPEs and narrow KAU

Yet, that HAEs are not necessary for understanding is precisely what some practitioners maintain and what contemporary philosophical discussions of theoretical modelling show. I examine here cases where HPEs provide understanding without actually explaining.

An oft-discussed example in the literature on HPEs is Schelling’s (1971, 1978) checkerboard model of residential segregation. According to Sugden (2000, 2), an economist himself, the checkerboard model “tells us something important and true about the real world”. Clark and Fosset (2008, 4109), both social scientists, consider that the model “was critical in providing a theoretical basis for viewing residential preferences as relevant to understanding the ethnic patterns observed in metropolitan areas”. Prior to the checkerboard model, social scientists believed that only strong discriminatory preferences—i.e., racism—could lead to residential segregation (Aydinonat 2007; Clark and Fossett 2008; Grüne-Yanoff 2009; Sugden 2000). The model showed that it was possible that preferences for not being in a minority status could also produce the same pattern of segregation. This result has proven to be very robust across changes of assumptions (Muldoon et al. 2012).

The checkerboard model is usually interpreted as having provided a HPE of residential segregation (e.g. Aydinonat 2007; Grüne-Yanoff 2013a; Kuorikoski and Ylikoski 2015; Weisberg 2013; Ylikoski and Aydinonat 2014). The model does not make any specific claim about the actual mechanism producing instances of residential segregation. More precisely, it is *not* a HAE of segregation since we do not know whether it explains any actual instance of that phenomenon. Instead, it answers a general how-possibly question, namely “how is it possible for segregation to happen in a city without collective preferences for segregation” (Weisberg 2013, 118-119)? Even though the model represents phenomena in a highly stylized manner and despite that the mechanism it depicts is not known to be actual, it still appears to provide causal knowledge about the phenomenon. Using the model, we know that if the mechanism were true, under suitable conditions residential segregation could be brought about. We know that it *could* actually depend on those factors or, conversely, that it does not necessarily depend on strong discriminatory preferences (Grüne-Yanoff 2009; Reiss 2008; Ylikoski and Aydinonat 2014). Knowing that some causal factors may bring about residential segregation improves our understanding of the phenomenon even though we do not know what actually causes it. It may do it in various ways. It can expand our ‘menu’ of possible explanations (Ylikoski and Aydinonat 2014). It can also license ‘what-if-things-had-been-different’ inferences about phenomena (Kuorikoski and Ylikoski 2015; Ylikoski and Aydinonat 2014). Or it can contradict impossibility theses scientists hold (Grüne-Yanoff 2009). All these accounts suggest the checkerboard model, interpreted as providing a HPE, can afford understanding of real-world phenomena.

Another widely discussed example in the literature on HPEs (e.g. Sugden 2009; Rice 2016; Rohwer and Rice 2013), this time at the intersection of biology and economics, is the ‘Hawk-Dove’ model (Maynard Smith and Price 1973; Maynard Smith and Parker 1976; Maynard Smith 1982) of animal competition. The Hawk-Dove is a game-theoretic model to study behaviour in situation of conflicts over a shareable resource. The model shows that restraint in contest between individuals of the same species benefit not only the species as a whole (the group), but also the individuals. Contrary to what was believed, a ‘limited war’ strategy can also benefit individuals’ fitness. It is not necessary to resort to group selection to explain that behaviour, individual selection is sufficient. Commenting on this type of modelling, Maynard Smith (1978, 52) said that “[t]he role of optimization theorizing in biology is not to demonstrate that organisms optimize. Rather, they are an attempt to understand the diversity of life”. In a similar vein, biologists Arnott and Elwood (2008, 529) note that our “understanding of this variation [of forms of contests for resources] was boosted by the application of game theory (Maynard Smith and Price 1973; Parker 1974), which examined how different strategies might be used by each contestant and how the winner is determined”. In the eyes of practitioners, the Hawk-Dove model contributed to our understanding of animal competition.

Like the checkerboard model, it also should not be viewed as a HAE. While providing a rationale for their research, Maynard Smith and Price wrote that “[a] main reason for using computer simulation was to test whether it is possible even in theory for individual selection to account for ‘limited war’ behaviour” (1973, 15). Their goal was not to provide a HAE of animal contest, but to study whether it could have evolved via individual selection. One philosophical interpretation of the Hawk-Dove model in line with the practitioners’ judgments is that it “produces some understanding of how individual selection could possibly lead to restraint in situations of animal conflict” (Rohwer and Rice 2013, 341). It does not answer a how-actually question, but instead aims to answer a how-possibly one, namely how individual selection could bring about restraint in combat (see also Rice 2016; Rohwer and Rice 2016). That this phenomenon may be the result of individual selection does not imply it is actually the case. Nonetheless, Rohwer and Rice (2013) argue that it affords understanding because it justifies the true belief that the restraint phenomenon is compatible with individual selection. According to them, this belief is relevant to answer the question why this phenomenon occurs.

Even though they do not satisfy the typical empirical external conditions associated with HAEs, both the judgement of practitioners and philosophical analyses of specific cases of theoretical modelling lead to the conclusion that HPEs can provide understanding. Proponents of the second tenet of narrow KAU could dispute this conclusion on two grounds. They could contend that HPEs are, in fact, explanations in the required sense of narrow KAU. Or, they could deny that HPEs can afford understanding. However, neither horn of the dilemma is readily available to them.

Firstly, it is implausible to regard all HPEs as being HAEs. Reydon (2012), for instance, argues that what Forber (2010) calls global HPEs are actually genuine explanations of type-level phenomena.^13^ Since the point of narrow KAU is precisely that *only* HAEs can provide understanding, this would be a successful way of defusing the claim that HPEs can afford understanding. Yet, the fact that some HPEs should perhaps rather be considered as HAEs does not exclude that others are genuine HPEs. As I already pointed out, Strevens (2013) stresses that an explanation must be externally correct, that is, it must contain a true explanans, in order to afford understanding. HPEs, by definition, do not satisfy these criteria because either the explanans or the explanandum is false or not known to be true. HPEs fail in some way or another to be HAEs. Furthermore, there are clearly cases—e.g., the ones discussed above—that do not satisfy the empirical conditions of adequacy. Insofar as empirical support is lacking for the explanans, models similar to the checkerboard or the Hawk-Dove examples can’t qualify as HAEs. So even though some HPEs could better be viewed as HAEs, not all can. For this reason, narrow KAU can’t account for them.

Secondly, rejecting the claim that some HPEs afford understanding implies that exemplary cases of theoretical modelling are epistemically suspect. The checkerboard and Hawk-Dove models have been very influential and, most importantly, have been considered to afford cognitive benefits in the form of understanding. That many contemporary philosophical accounts as well as practitioners consider some HPEs afford understanding is strong evidence that they actually do so. As far as practice is correctly described, the burden of proof should be on those philosophical accounts that want to deny HPEs can afford understanding, not on practitioners.

In a related but separate discussion, Fumagalli (2015, 2016) argues that both the checkerboard and the Hawk-Dove models, interpreted as ‘minimal models’—that is, models that supposedly lack any representational features—can’t justify a change of confidence in necessity or impossibility theses (see Grüne-Yanoff 2009). The checkerboard model, for instance, affects our confidence in the thesis that only strong discriminatory preferences can bring about residential segregation. Fumagalli may be right as far as minimal models thus defined are concerned. But this does not imply that HPEs need be minimal models. Actually, the mistake seems to rest in regarding the checkerboard and the Hawk-Dove models as minimal. The checkerboard model may afford understanding precisely in virtue of *some* similarity or resemblance between the world and the model (Sugden 2009; Ylikoski and Aydinonat 2014). To consider that the mechanism depicted by the checkerboard model is causally possible indeed appears to require at least a minimal assessment of its similarity with the actual world.

One could nevertheless argue, as Resnik (1991) and Reydon (2012) do, that HPEs are in fact HAEs or merely serve heuristic purposes because they are not HAEs. But what is at stake is not whether HPEs are a species of explanations or not, but whether they afford understanding. Reydon, for instance, does not specifically address this issue. The fact that HPEs lack full empirical support does not necessarily imply that they can’t afford understanding. HPEs may sometimes serve heuristic purposes, but other times they may also afford understanding. The checkerboard model does both. It suggests a novel empirical hypothesis that can orient future research, while also allowing to answer various questions about residential segregation. The two functions are not necessarily mutually exclusive.

It might be the case that scientists are sometimes too optimistic about results from HPEs or that they mistake some HPEs for HAEs. There might thus be cases of HPEs that do not improve our understanding. However, such negative readings do not imply that HPEs can’t, out of principle, afford understanding. Descriptively, denying this capacity to HPEs is infelicitous as actual practitioners consider they afford understanding. However, the normative point may still hold, viz. that practitioners are in fact mistaken. That said, proponents of narrow KAU would need to offer a plausible argument for why we should consider they are indeed mistaken. Arguments of that kind are currently lacking.

If HPEs may afford understanding, as it is plausible they sometimes do, then narrow KAU faces a serious objection: it appears that having an explanation, in the sense of a HAE, is not necessary for understanding. Whereas HAEs of course afford understanding, HPEs may also.

## Broad KAU

The two tenets of narrow KAU are untenable. First, the literature on non-causal explanations provides good reasons to believe that causal knowledge is not necessary for understanding. Second, as I have argued, there are cases of theoretical modelling that do not provide explanations and yet, according to practitioners and philosophers, afford understanding. This indicates that having an explanation is also not necessary for understanding.

Therefore, if we want our epistemology of understanding to apply to cases of theoretical modelling, then we need an alternative to narrow KAU, in general, and to its second tenet, in particular. *Broad* KAU, I contend, provides an alternative epistemology of understanding that fulfils the desiderata set forth in the introduction. In particular, accounting for scientific practice should not come at the cost of blurring the difference between illusory and genuine understanding. It should also make salient the relationship between explanation and understanding.

Broad KAU 1) broadens the knowledge—i.e., not only knowledge of causes—that affords understanding and 2) broadens the ways this knowledge can be acquired. Broad KAU thus challenges that causal knowledge and that having an explanation are necessary for understanding. More formally, broad KAU contradicts narrow KAU in two ways:

1. It asserts that causal knowledge is not necessary.
2. It asserts that having an explanation is not necessary.

One fruitful way of advancing towards a more formal characterization of broad KAU is to look at Reutlinger’s (2016) theory of counterfactual explanation. This is because it already embraces one element of broad KAU, namely it welcomes non-causal knowledge. Reutlinger’s theory aims at capturing the ‘common element’ of causal and non-causal types of explanation without necessarily being tied to an interventionist interpretation of counterfactuals. Reutlinger’s strategy is to stay as close as possible to the Woodwardian (2003) spirit of causal explanation, while making room for non-causal generalizations to serve as explanantia. An important motivation of his is precisely to accommodate mathematical explanations such as the widely discussed Königsberg bridges case (see, e.g., Pincock^2007^). According to Reutlinger, a relation between an explanans and an explanandum is explanatory iff it satisfies the following conditions (2016, 737):

**Veridicality condition**: Generalizations *G*_1_,…, *G*_*m*_, the auxiliary statements *S*_1_,…, *S*_*n*_, and the explanandum statement *E* must all be (approximately) true or be well confirmed.
**Implication condition**: *G*_1_,…, *G*_*m*_ and *S*_1_,…, *S*_*n*_ logically entail *E* or a conditional probability *P*(*E*|*S*_1_,…, *S*_*n*_).
**Dependency condition**: *G*_1_,…, *G*_*m*_ support at least one counterfactual between *S*_1_,…, *S*_*n*_ and *E*.

Since Reutlinger’s theory allows for non-causal generalizations, it already incorporates the first tenet of broad KAU. The conditions he states are those that explanations (i.e., HAEs) must satisfy. Accordingly, his theory leaves out the second tenet of broad KAU, viz. that an explanation is not necessary for understanding.

The first step that will allow us to filter out understanding from explanation is by identifying in virtue of what explanations provide understanding. To put it in Lipton’s (2009) terms, we have to separate the benefit explanations provide—understanding—from the explanations themselves. Reutlinger is not explicit about this, but since his theory is an extension of Woodward’s (2003), we can find indications there. For Woodward, explanations provide understanding because they convey information that is relevant to answering ‘what-if-things-had-been-different’ questions about a phenomenon of interest. One understands when one obtains information about counterfactual dependence that allows to answer these questions. We thus see that what is key to understanding is the *information* some propositions provide, information that is closely related to the satisfaction of the dependency condition.

Broad KAU expands on the idea that it is essentially information about counterfactual dependence that contributes to understanding, regardless of whether it is causal or not, and, crucially, regardless of whether it is obtained through an explanation or not. Reutlinger’s theory already accommodates non-causal dependence by modifying the dependency condition. Having an explanation implies that certain relations of dependence actually obtain. This information that explanations provide allows to answer ‘what-if-things-had-been-different’ questions. But is it possible to answer some ‘what-if-things-had-been-different’ questions about a phenomenon even if the relations of dependence are not actual? The challenge for broad KAU, therefore, is to show that having an explanation is not necessary for satisfying the dependency condition. Put differently, how could the dependency condition, which appears to be essential for understanding, be satisfied without the veridicality condition?

Reutlinger proposes an account of explanation and explanations are usually taken to be factive, i.e., they give true accounts of the facts. The function of the veridicality condition is precisely to ensure the factivity of explanation. False generalizations would not explain an explanandum and true generalizations would not explain an explanandum that is known to be false. For a set of propositions to count as an explanation, both the explanans and the explanandum must be true. But what if the veridicality condition is not satisfied? What if the generalizations are false or not known to be true? What if the explanans or explanandum are merely possible? Put differently, what if we have a HPE? According to Reutlinger’s account, this would imply the relationship is not explanatory. However, if we accept the compelling evidence that HPEs may provide understanding despite the fact that they contain false explanantia or explananda, then this suggests that the veridicality condition is not necessary for understanding. Since we can obtain understanding from false explanantia or explananda, the veridicality condition may be necessary for explanation, but not for understanding.

This indicates that we can disentangle the condition for actual explanation in Reutlinger from the core constituents that specifically concern understanding. We can achieve this result, I submit, simply by amending the veridicality condition. Accordingly, we can modify the theory of explanation to achieve a theory of understanding. I propose that the relationship between an explanans and an explanandum affords understanding iff:

**Possibility condition**: The generalizations *G*_1_,…, *G*_*m*_, the auxiliary statements *S*_1_,…, *S*_*n*_, or the explanandum statement *E* are (im)possible according to the relevant modal interpretation and epistemic goal.
**Implication condition**: Generalizations *G*_1_,…, *G*_*m*_ with the auxiliary statements *S*_1_,…, *S*_*n*_ logically entail *E* or a conditional probability *P*(*E*|*S*_1_,…, *S*_*n*_).
**Dependency condition**: *G*_1_,…, *G*_*m*_ support at least one counterfactual between *S*_1_,…, *S*_*n*_ and *E*.

In other words, I propose to amend the veridicality condition for what I call the *possibility condition*.^14^ Essentially, it relaxes the explanatory requirement that the explanans and explanandum be actual. What is actual is possible, but what is possible is not necessarily actual. Explanations require that all its constituents are actual, but not understanding.

This fits very well with accounts of HPEs that view them as describing “how a set of parts and activities *might be organized* such that they exhibit the explanandum phenomenon” (Craver 2006, 361, my emphasis; see also Verreault-Julien 2018). They show how an event could possibly occur or how known processes can lead to different outcomes. This, in turn, affords understanding of real-world phenomena. For instance, the checkerboard model exhibits a possible causal mechanism—possible causal generalization—that can bring about residential segregation, which is an actual phenomenon. However, the model itself does not explain residential segregation because we do not know whether it is actually that mechanism that produces segregation. Yet, the checkerboard model affords understanding by virtue of showing how it *could* be brought about. It supports counterfactuals of the form ‘If individuals had not strong discriminatory preferences, then residential segregation could still occur’. More generally, it allows to make various counterfactual inferences about the phenomenon of residential segregation (Kuorikoski and Ylikoski 2015; Ylikoski and Aydinonat 2014). Despite the fact that it does not satisfy the veridicality condition, it does satisfy the possibility and the dependency conditions. If it were the case that preferences for not living in a minority status cause the phenomenon, then we would have an explanation. We would also of course understand. But we can also understand even when we lack an explanation insofar as the dependency condition is satisfied.

The “(im)possible according to the relevant modal interpretation and epistemic goal” clause within the possibility condition makes explicit that 1) there are various ways we can deem a proposition to be (im)possible (Lange 2009; Kment 2012) and 2) that understanding is achieved on the background of a specific epistemic goal. Many HPEs, for instance the checkerboard model, display possible *causal* dependence. However, other cases may appeal to different modalities (e.g., epistemic, nomic, metaphysical, etc.). In mathematical explanations, the relevant modality is mathematical or logical. The truth or falsehood of possibility claims is reached on the background of the suitable facts, depending both on the modality and on the epistemic goal. To say that something is logically possible does not imply that it is also causally possible. The same constraints do not apply. For the purpose of scientific understanding, causal, epistemic, or nomic possibility are perhaps the most relevant types. Broad KAU, however, does not rule out a priori the sorts of possibilities or relations of dependence that may afford understanding.

To make this clearer, let us use an example discussed by Strevens (2013), that of the young earth creationists. They purportedly explain the formation of the Grand Canyon by citing one massive flood occurring over a short period of time. The flood would have laid down most of the different rock layers and the flood would have dug the canyon itself. Strevens says that he “cannot think of any conversational context in which it is correct to say, without frantic hedging, that the young earth creationists understand the formation of the Grand Canyon” (2013, 513). That they do not understand follows from Strevens’s requirement that one needs to grasp a correct explanation in order to understand. The great flood explanation is false, i.e., its explanans is not true in the sense that it is not actual. The Grand Canyon was actually formed by other geological processes, namely by sediment accumulation, plate movement, and slow erosion. Recent evidence suggests large parts of the canyon may be as old as 70 million years (Flowers and Farley 2012), a far cry from the young creationists’ claim.^15^

But does relaxing the veridicality condition imply that comparable cases may satisfy the possibility condition and thus afford understanding? If so, it may imply that the possibility condition is too liberal since utterly wrong-headed HPEs could afford understanding. Since one desideratum of an account of understanding is to allow demarcation of illusory from genuine understanding, this would be an unwelcome implication of broad KAU. Again, offering a descriptively adequate epistemology of understanding should not make it too easy to obtain.

I agree with Strevens that the young earth creationists’ great flood explanation does not afford understanding. Here, it is important to take into consideration what is the relevant modal interpretation for a given epistemic purpose. This allows to clarify how exactly a HPE contributes, or not, to understanding. If one asks ‘How possibly could have the Grand Canyon been formed?’, the question can most appropriately be interpreted as a request for causal information about the Grand Canyon. One wants to know how the Grand Canyon could have possibly been causally formed. Hence, not only is the young earth creationists’ great flood explanation actually false, but according to what we know about geological processes, this could *not* have happened the way the young earth creationists claim. In other words, the great flood explanation does not qualify as being a HPE and is not an appropriate answer to the how-possible question above.^16^

By that, I mean that the causal generalization linking the flood to the canyon is not only inconsistent with what we know about the causal history of this specific case, but also with other more general causal facts. It conflicts with the fact that geological processes forming canyons operate over long periods of time, not during one year. And while floods may produce certain geological formations (e.g., the Channeled Scablands in the US, see Baker and Bunker 1985; Waitt 1980), the floods are local, not global. It also contradicts scientific facts about the age of the earth and of the Grand Canyon. Or it can hardly account for the presence of fossils in the different rock layers. What makes the young earth creationists’ explanation an inadequate answer to the how-possible question is not so much the fact that floods can play a causal role, but the precise way they claimed it happened and how inconsistent it is with everything else we know about geological processes. In a nutshell, it just could *not* have happened the way the young earth creationists claim. The great flood explanation thus does not fulfil the possibility condition since the how-possibly request calls for information about causal possibility, which the young earth creationists do not provide.

It also does not satisfy the dependency condition because the generalization linking floods and the Grand Canyon does not support the right kind of (true) counterfactuals. For instance, the young earth creationists’ explanation implies that the following counterfactual is true: ‘Had there been a great flood, the Grand Canyon would have been formed as a result’. However, that counterfactual is false. Perhaps if other geological processes would also have been sufficiently different the flood could have formed the canyon, but this qualification is not part of the young earth creationists’ story. Absent changes to these other processes, the flood would not have brought about the Grand Canyon. It is at best unclear what sort of ‘what-if-things-had-been-different’ questions the great flood explanation may answer.

All that said, since the possibility condition allows for *im* possible explanantia and explananda, does this mean the great flood generalization would, on reflection, afford understanding? It could, but only in a specific set of circumstances. Let us suppose that we did not know that a flood during a very short period of time could have dug the canyon and laid down the rock layers, but that we did know that the actual explanation involved erosion, sediment accumulation, and plate movement. One question we may ask ourselves is whether other causes, like the flood, could have brought it about. We may thus build a model or simulation in which we try to generate the Grand Canyon with an intense flood as the main causal driving force. In order to achieve this result, we would most probably need to assume very different geological conditions and processes than the ones currently existing. In doing so, we would thus learn about the contingency, or necessity, of the actual explanation. We would also learn to what extent the actual world would need to be different for the flood to have the capacity to form the canyon. As Weisberg argues in discussing similar examples of impossible explananda, modelling the impossible is a sound scientific practice which may afford understanding.

Why should theorists who are primarily interested in studying what is actual try to understand what isn’t actual? The answer to this question cuts deep into the heart of theoretical practice: Theorists ultimately aim to partition the space of possibilities. They aim to understand what is possible, what is impossible, and why (Weisberg 2013, 128).

What this shows is that whether a given HPE affords understanding or not depends on what is exactly achieved and on the context of enquiry. Whereas the young earth creationists’ specific ‘explanation’ brings nothing to the table in terms of understanding, we can imagine slightly different scenarios where investigating the conditions under which a flood could have formed the Grand Canyon would yield understanding. We understand better the Grand Canyon if we establish, say, that it is impossible to generate it with known initial conditions and a great flood as a possible process. Or that it is possible that a flood forms the Grand Canyon, but only if the world would have been a very different place. These generalizations may for instance support the counterfactual that ‘Had there been a flood, the canyon would not have been formed as a result’. The crucial difference between this illustrative case and the young earth creationists’ one is that the latter does not identify relevant possible causal generalizations and that these generalizations do not support counterfactuals.

Another way of illustrating this point is to briefly consider the cases of phlogiston theory or Ptolemaic astronomy. As I said in Section 3, both theories rely on false generalizations and therefore do not explain. Looking at these cases with our contemporary eyes, shall we say that they afford understanding?^17^ Our account of understanding should view these cases as offering at best a limited understanding of nature. As with the young earth creationists’ case, much depends on what we take the relevant ‘what-if-things-had-been-different’ questions to be. Both theories are famously unable to answer some questions that concern the phenomena they are supposed to explain. For instance, phlogiston theory has trouble supporting counterfactuals concerning the combustion of metals since some gain weight instead of losing it. Moreover, not only are their generalizations false, they provide a wrong-headed picture of the world. So perhaps we might say that they depict *im* possible explanantia—e.g., the earth at the centre of the universe—, but whether phlogiston theory or Ptolemaic astronomy would satisfy the possibility and dependency conditions again depends on the epistemic goal and what sort of counterfactuals are under investigation. For example, one might be interested in exploring what difference it would make if the earth were at the centre of the universe. This may help understand why the world is the way it is. However, that is a different epistemic goal than what Ptolemaic astronomy purported to do.

My proposed account of the conditions under which the relationship between an explanans and an explanandum affords understanding, therefore, has the resources to discriminate between genuine and specious cases of understanding. While broad KAU seeks to be as accommodating as possible in order to reflect actual scientific practice, it also imposes limits on what can count as affording understanding. The possibility and dependency conditions provide criteria according to which we can assess whether or not a relationship affords understanding.

One final objection may be the following: does discarding the veridicality condition imply that understanding is not factive? Reutlinger’s (2016) veridicality condition originates from the usual requirement that explanations are factive. We may not need factive explanations for understanding, but we still want understanding to be factive. How can we have factive understanding without (factive) explanation? We can because the function of the veridicality condition, as it is formulated, is simply to ensure the relationship could be part of an actual explanation, not that it would afford understanding. An explanation is constituted by an actual explanandum and an actual explanans. A possible or false explanans does not explain an actual explanandum. That being said, a relationship that does not satisfy the veridicality condition may afford understanding. Indeed, it should not obscure the fact that generalizations can still support counterfactuals, and thus satisfy the dependency condition, even if the explanans or the explanandum are merely possible. This is, in fact, the determining component of understanding. As we have seen above, it is information that allows to answer ‘what-if-things-had-been-different’ questions that is relevant for understanding (Grimm 2010; Woodward 2003). Explanations provide that kind of information, as HPEs can do. We can truthfully answer some ‘what-if’ questions even when we lack the actual explanation. For example, the checkerboard model shows that residential segregation could, in suitable conditions, be brought about if individuals had certain preferences. Since we consider the mechanism to be causally possible, we can use that information to answer ‘what-if’ questions. It, therefore, satisfies the dependency condition. To know that an explanandum may depend on a possible explanans is genuine knowledge of dependence and is, in that sense, factive. Likewise, that a possible explanans supports at least one counterfactual implies that we can evaluate its truth. Understanding is therefore still factive because it relies on matters of facts about possibility and counterfactuals. One would not understand without knowledge of possibility or without truthful assessment of counterfactuals. Knowing that something is possible responds to facts about possibility. This makes understanding factive. Broad KAU therefore does not necessarily come at the cost of having to reject the factivity of understanding.^18^

## Conclusion

Neither tenet of narrow KAU is necessary. In other words, understanding does not depend on causal explanation. Amending Reutlinger’s (2016) theory of counterfactual explanation as suggested above yields a sound basis for an epistemology based on broad KAU. Upholding narrow KAU comes at the price of having an epistemology of understanding that can’t appraise neither descriptively nor normatively current scientific practices like theoretical modelling. We do not have to pay that price. Broad KAU can easily accommodate the view according to which some HPEs afford understanding even though they do not actually explain. Broad KAU thus has a lot of appeal since it coheres with the actual conduct of science. It therefore fulfils an important desideratum of an account of understanding, namely that it should not regard vast areas of science as being mistaken about what they achieve. That said, broad KAU’s flexibility does not come at the cost of blurring the distinction between illusory and genuine understanding. The possibility condition I propose, while accommodating, is not a free ticket to understanding.

## Footnotes

1 Whether narrow KAU states sufficient conditions for understanding is a different question. While it appears prima facie plausible, here I remain agnostic over this issue.

2 The recent literature on understanding, while not examining non-causal cases in detail, also rejects the first tenet of narrow KAU (e.g. de Regt 2017; Greco 2014; Grimm 2014; Khalifa^2017^).

3 Woodward’s insight, in particular, has recently received some attention (e.g. Grimm 2010; Pincock 2015; Reutlinger 2016).

4 See, e.g., Skow (2014) or Strevens (2018) for views that challenge this liberal consensus.

5 See also Strevens (2008, 3).

6 Khalifa (2013c) argues that the crucial cognitive ability involved in understanding, which we may call grasping, is the ability to conduct a reliable explanatory evaluation. See also Khalifa and Gadomski (2013).

7 There is also a debate over whether understanding, unlike knowledge, can be ‘lucky’ (see, e.g, Hills 2016; Khalifa 2013c; Morris 2012; van Riel 2016; Rohwer 2014). More minimally, we could say that what is required is to have true beliefs about the explanation. Nothing here hinges on how this question is settled.

8 Strevens (2013) explicitly disagrees with some of Lipton’s examples, but says nothing about the general approach. See Khalifa (2013b) for an in-depth analysis of Lipton’s strategy, its success, and limitations.

9 See Verreault-Julien (2018) for a detailed analysis of the different accounts of how-possibly explanations.

10 What I say here is in principle orthogonal to the scientific realism debate. As Khalifa (2011) argues, to say that explanation is necessary for understanding does not imply the factivity of the explanans. It only requires the explanation to be correct according to some metric, e.g., empirical adequacy. A HPE thus can simply be an explanation that is not correct or not known to be correct. But, of course, many accounts require the (approximate) truth of the explanans for understanding (e.g. Strevens 2013; Trout 2007; Woodward^2003^).

11 There is also a distinction to be made between understanding *with* a theory or model, also sometimes called objectual understanding (see Khalifa 2013a; Kvanvig 2003), and understanding *why*. We are here only concerned with the latter. It is thus possible to understand combustion *with* phlogiston theory while not understanding *why*. To understand *why* some externals conditions of adequacy need to be fulfilled.

12 For instance, between the potential and true explanations, Hempel (1965, 338) also proposed the ‘more or less strongly supported or confirmed’ explanation.

13 Forber (2012) claims nothing really hinges on that distinction because for him global how-possibly explanations are a kind of explanations.

14 Reutlinger and others (Reutlinger 2017; Reutlinger et al. 2018) themselves suggest that HPEs do not satisfy the veridicality condition. The possibility condition I put forward is a novel proposal.

15 The age of the Grand Canyon is an on-going debate. Scientific advocates of a ‘young’ canyon date its origin to about 5–6 million years. In any case, we are still far from the young earth creationists who claim the earth was formed between five and ten thousand years ago.

16 Of course, young earth creationists would most probably disagree with this statement. The flood causing the formation of the Grand Canyon is consistent with their other background beliefs. Here I am assuming that the geological sciences provide reliable empirical information.

17 I want to set aside the question whether phlogiston theory, for instance, increased understanding of its contemporaries. This is out of the scope of this article.

18 Some (e.g. Elgin 2007; Kvanvig 2003) maintain that understanding is non-factive. Whether it is or not has no direct implication for the account just presented.
